# Prevalence of G6PD deficiency and *Plasmodium falciparum* parasites in asymptomatic school children living in southern Ghana

**DOI:** 10.1186/s12936-016-1440-1

**Published:** 2016-07-26

**Authors:** Linda Eva Amoah, Akua Opong, Ruth Ayanful-Torgby, Joana Abankwa, Festus K. Acquah

**Affiliations:** 1 Immunology Department, Noguchi Memorial Institute for Medical Research, College of Health Sciences, University of Ghana, Legon, Accra, Ghana; 2 Ghana Health Service, Ministry of Health, Accra, Ghana

**Keywords:** Malaria, G6PD, *Plasmodium falciparum*, Primaquine, Genotyping, RFLP, ELISA

## Abstract

**Background:**

Glucose-6-phosphate dehydrogenase (G6PD) deficiency is an X-linked genetic disorder that results in impaired enzyme activity. Although G6PD deficiency is globally distributed it is more prevalent in malaria-endemic countries. Several mutations have been identified in the G6PD gene, which alter enzyme activity. The G6PD genotype predominantly found in sub-Saharan Africa is the G6PDB (G6PD_376A_) with (G6PD_376G_) and G6PDA- (G6PD_376G/202A_, G6PD_376G/542T_, G6PD_376G/680T_ and G6PD_376G/968C_) occurring at lower frequencies.

**Aim:**

The aim of this study was to identify the prevalence of G6PD deficiency and asymptomatic *Plasmodium falciparum* carriage in children living in southern Ghana and determine whether G6PD deficiency influences asymptomatic carriage of *P. falciparum* parasites.

**Methods:**

Blood samples were obtained once a month from 170 healthy Ghanaian school children aged between 5 and 12 years from Basic schools in two communities Obom and Abura with similar rainfall patterns and malaria peak seasons. G6PD enzyme activity was assessed using the qualitative G6PD RDT kit (AccessBIO). G6PD genotyping and asymptomatic parasite carriage was determined by PCR followed by restriction fragment length polymorphism (RFLP) of DNA extracted from dried blood spots.

**Results:**

The only sub-Saharan G6PD A- allele detected was the A376G/G202A found in 12.4 % (21/170), of the children and distributed as 4.1 % (7/170) A-, 1.8 % (3/170) A-/A- homozygous deficient males and females and 6.5 % (11/170) A/A- and B/A- heterozygous deficient females. Phenotypically, 10.6 % (15/142) of the children were G6PD deficient. The asymptomatic carriage of *P. falciparum* by PCR was 50, 29.4, 38.2 and 38.8 % over the months of February through May 2015, respectively, and 28.8, 22.4, 25.9 and 5.9 % by microscopy during the same periods.

**Conclusions:**

G6PD deficiency was significantly associated with a lowered risk of PCR-estimated asymptomatic *P. falciparum* carriage in children during the off peak malaria season in Southern Ghana.

**Electronic supplementary material:**

The online version of this article (doi:10.1186/s12936-016-1440-1) contains supplementary material, which is available to authorized users.

## Background

Malaria is a deadly disease caused by five *Plasmodium* species with *Plasmodium falciparum* being the most lethal. *Plasmodium falciparum* caused an estimated 438,000 deaths globally in 2015 [1]. The heaviest burden of malaria, according to the World Health Organization (WHO), is in Africa where an estimated 395,000 malaria deaths was recorded in 2015 [1].

G6PD deficiency (G6PDd) is one of the most common human genetic enzyme defects [2], affecting over 400 million people. Although this enzymopathy is globally distributed, it is more prevalent in the tropics and sub-tropics, especially in malaria-prone countries [3]. This X-linked genetic condition is characterized by reduced G6PD enzyme activity, which can remain asymptomatic. Red blood cells obtain reduced glutathione (GSH) solely from the G6PD/reduced nicotinamide adenine dinucleotide phosphate (NADH) pathway. The deficiency makes red cells more susceptible to oxidative haemolysis that can be triggered by certain drugs, such as primaquine (PQ) and other 8-amino quinolone drugs [4]. Glucose-6-phosphate dehydrogenase (G6PD) and a number of other human genetic traits including sickle cell anaemia and related haemoglobinopathies are predominantly found in populations living in malaria endemic countries and have been suggested to provide the host protection from severe forms of malaria [5–8] and asymptomatic malaria [9]. A number of genetic traits that protect the host against severe forms of malaria have recently been found to promote the development of the sexual transmissible stages of the parasite, and individuals with these traits serve as reservoirs for malaria transmission [10] or alter the acquisition of anti-parasite antibodies [11].

G6PD deficiency can be determined using a number of approaches, including, quantitative tests, which measure precise enzyme activity, qualitative tests which classify enzyme activity as normal or deficient and genetic tests, which identify gene mutations [12–19].

Over 400 G6PD variants have been identified [4] and the polymorphisms are predominantly defined to specific geographic locations [20]. In sub-Saharan Africa, the predominant G6PD variants are B, A and A-, with frequencies greater than 1 %. The G6PD B variant has the 376A cDNA sequence and is classified as possessing normal enzyme activity. The non-deficient G6PD A variant has been shown to possess about 85 % of the activity of the normal enzyme and is classified as possessing normal activity carries a cDNA mutation A376G, which translates into amino acid N126D. The sub-Saharan G6PD A- variants carry the G6PD A backbone with an additional single mutation, the most common A- variant has the A376G/G202A cDNA mutation. G6PD A- been suggested to possess 10 % of normal enzyme activity in their red blood cells though their white blood cells maintain 100 % normal enzyme activity [21] Additional A- variants peculiar to sub-Saharan Africa include the A376G/A543T, A376G/G680T and A376G/T968C [19].

Global efforts to achieve malaria elimination have lead the WHO to recommend PQ, the only WHO certified anti-gametocyte drug, to be incorporated into malaria treatment regimens in selected countries to reduce malaria transmission [22]. Although low-dose PQ has been found to be safe for use in G6PDd individuals [23, 24], a number of other reports have found PQ to cause dangerous side effects in G6PDd individuals [25, 26] making it necessary to monitor the prevalence of G6PD as well as determine possible effects this trait has on malaria in Ghanaians.

## Methods

### Study site and sampling

Abura Dunkwa, also known as Abura, is the district capital for Abura-Asebu-Kwamankese district of the Central Region with a rural population of 31,768 for children under 14 years of age [27]. Obom is in the Ga south municipality of the Greater Accra Region with a rural population of 22,368 for children under 14 years of age [28]. Abura (Cape Coast) is an area of seasonal malaria transmission and Obom (Accra) is an area of perennial malaria transmission, both of which have the peak malaria season between June to August. Inhabitants of these regions have different ethnicities. Although a total of 200 school children aged from six through 12 years were recruited for this study, 30 missed at least one of the four sampling days. Out of the 170 children, 150 were randomly selected for phenotypic analysis of G6PD enzyme activity.

### Microscopic estimation of malaria parasite

Thick and thin blood smears as well as dried filter paper blood spots (DBS) were each made from a drop (~50 μl) of finger-prick blood. The slides were air-dried, the thin smear fixed in methanol and both smears stained with 10 % Giemsa for 15 min. The stained slides were subsequently air-dried and viewed under 100X oil immersion microscope. Two independent microscopists read the slides. Parasitaemia was determined as the % of malaria parasites observed per 200 white blood cells (WBCs).

### DNA extraction from dried blood spots

DNA and antibodies were extracted from the DBS [29]. Briefly, two 3 mm disks were punched from the filter paper blood blots into a 2 ml microcentrifuge tube. The tube was then filled with 1120 μl of 0.5 % saponin and left to shake overnight at room temperature. The saponin supernatant containing the eluted antibodies was decanted and saved for the ELISAs. The disks were washed for 30 min in ice cold PBS at 4 °C. The wash solution was decanted and the tubes filled with 150 μl of 6 % chelex in PBS. The tubes were then heated at ~99 °C for 30 min with a quick vortex and centrifuge every 10 min. The heat extracted DNA was aliquoted into a new tube and either used immediately or stored at 4 °C for use within 2 weeks.

### *Plasmodium falciparum* genotyping

MSP2 and/or GLURP genotyping was performed on the extracted gDNA to simultaneously determine parasite prevalence and diversity. The WHO malaria parasite genotyping protocol (23) was followed with slight modifications. PCR reactions were carried out in 15 μl volumes for both the primary and nested reactions. Briefly the 200 nM M2-0F and M2-0R primers were used to amplify 4 μl of extracted DNA using One Taq polymerase (NEB). The nested reaction was carried out using 1 μl of the primary PCR product with 200 nM each of the combination of S1Fw/N5rev for the 3D7 type alleles or S1Fw/M5rev for the FC27 type alleles. For GLURP, the G-F3 and G-F4 primer pair was used for the outer PCR reaction and the G-NF and G-F4 primer pair used for the nested inner reaction. All the PCR fragments and the digested products were viewed under UV after resolving on a 2 % agarose gel containing 0.5 µg/ml ethidium bromide.

### G6PD enzyme activity

Rapid detection of enzyme activity was determined using the G6PD rapid diagnostic test kits (Access BIO, USA) according to manufacturer’s instructions. Briefly, the finger was pricked with a lancet provided in the kit and the blood spotted on to the sample pad of the test cassette. Two drops of buffer was applied to the sample pad and after 15 min the colour of the sample pad was noted. A purple membrane indicated normal enzyme activity whilst a white membrane indicated enzyme deficiency.

### G6PD genotyping

PCR was performed on DNA extracted from the DBS as previously reported [30]. The A376G polymorphism was analysed in each DNA sample using PCR followed by digestion with 1 unit of FOKI restriction enzyme under manufacturer-recommended conditions in a thermal cycler. Only those samples that were 376G and thus produced a digested product were further analysed for three other sub-Saharan African cDNA mutations, G202A, G680T and T968C by similar restriction fragment length polymorphism. The G202A PCR amplicon was digested with NlaIII restriction enzyme, the G680T amplicon was digested with BstNI restriction enzyme and the T968C amplicon digested with NciI restriction enzyme for 1 h at 37 °C. All the PCR fragments and the digested products were viewed under UV after resolving on a 2 % agarose gel containing 0.5 µg/ml ethidium bromide.

### Statistical analysis

Microsoft excel and SPSS 22.0 (IBM Corp) were used for all data analysis and to generate the frequency and cross tabulation tables. Frequency of parasite carriage was calculated as the (Additional file 1) sum of the number of times a child was observed to be parasite positive over the four time points. SPSS linear regression was used with model fit analysis to identify significant correlations between frequency of parasite carriage estimated by microscopy and PCR (dependent variables) and G6PD genotype and phenotype (independent variables). The relationship between G6PD genotype and phenotype was obtained using crosstabs descriptive statistics. Statistical significance was defined as *p* value  ≤0.05.

## Results

### Study site

**Fig. 1:**
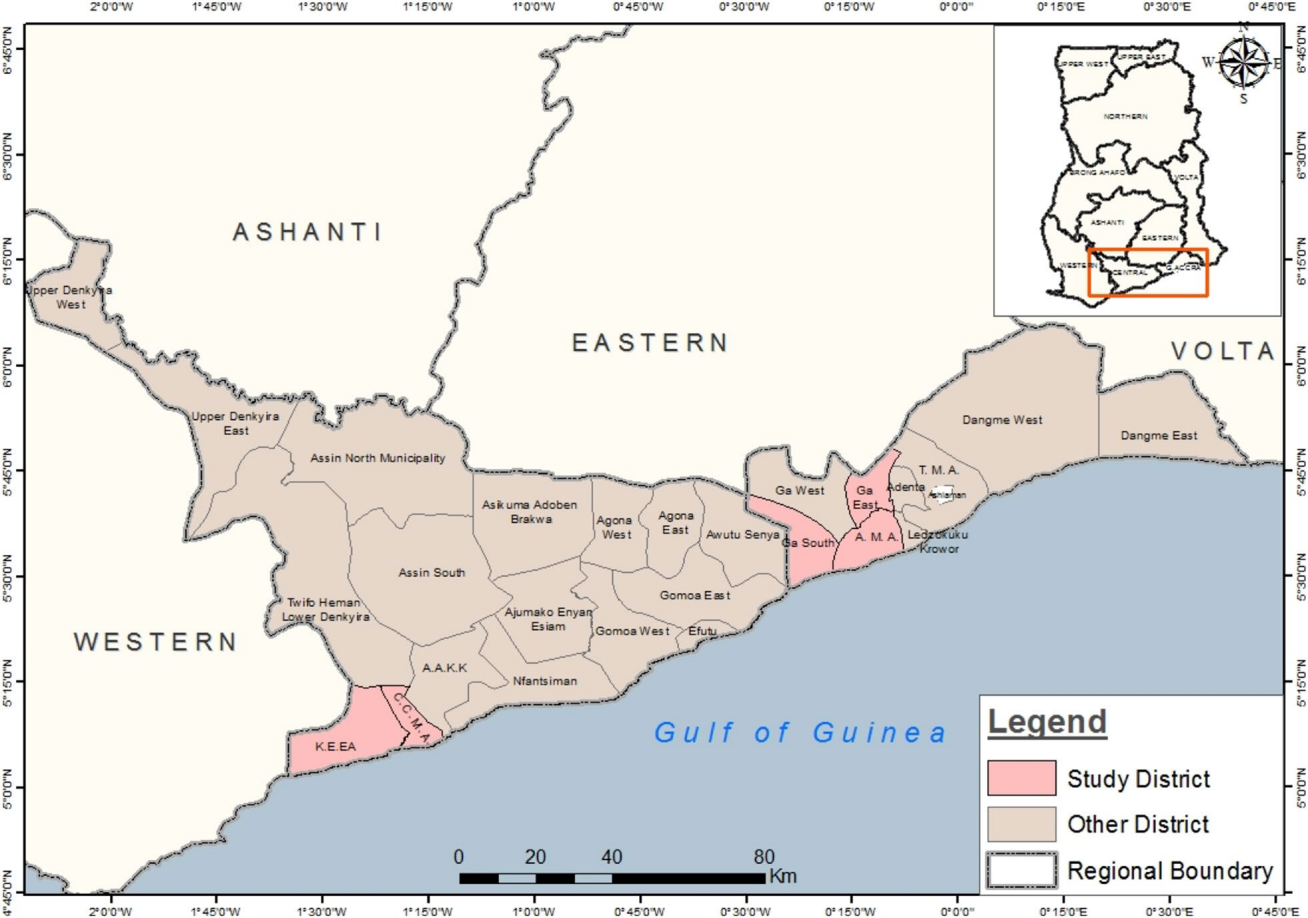
Map showing the location of study sites. Children were selected from four schools, two each in Obom in the Ga South municipality of the Greater Accra Region and Abura, in the Cape Coast municipality of the Central Region. Both sites lie on the southern coast of Ghana

The study utilized blood samples collected from a longitudinal survey of children living in southern Ghana (Fig. 1) from February through May 2015. Two hundred study participants were recruited but the data presented analysed samples from 170 children who were available for all four blood collection visits. The mean age of the children was 9.12 ± 0.13 however 45 % of the children were either nine or 10 years old. Fifty-four percent (92/170) of the children were female (Table 1), however the ratio was slightly increased to 56 % (80/142) in the subset used for the G6PD phenotype analysis. None of the children were symptomatic for malaria during any of the sampling visits.

**Table 1:** Distribution of G6PD genotype and phenotype by sex

	Deficient (P)	Deficient (G)	Heterozygous deficient (G)
Female	5.6 % (8/142)	1.8 % (3/170)	6.5 % (11/170)
Male	4.9 % (7/142)	4.1 % (7/170)	0 % (0/170)

Deficient (P), deficient G6PD enzyme activity; Normal (P), normal G6PD enzyme activity; Deficient (G), deficient genotype (A- and A-/A-); Heterozygous deficient (G), female heterozygous deficient (A/A- and B/A-); Normal (G), normal genotype (A, B, A/A, A/B and B/B)

### G6PD phenotype

Out of the 150 children sampled with the G6PD RDT, 8 gave invalid results. From the 142 valid test results, 10.6 % (15/142) were branded G6PD deficient, with boys representing 47 % (7/15) of the total G6PDd population (Table 1). Ninety-one percent and 85 % of boys and girls respectively carried normal G6PD genotypes while 89 and 90 % of boys and girls were characterized as possessing normal G6PD enzyme activity (Table 1).

### G6PD genotype

The only G6PD A- genotype identified was A376G/G202A, which was identified in seven hemizygous G6PDd (A-) males, 11 heterozygous G6PDd females (B/A-, A/A) and three homozygous G6PDd (A-/A-) females, representing 4.1, 6.5 and 1.8 % of the 170 school children respectively (Table 1).

### Microscopic and PCR estimation of parasite carriage

**Fig. 2:**
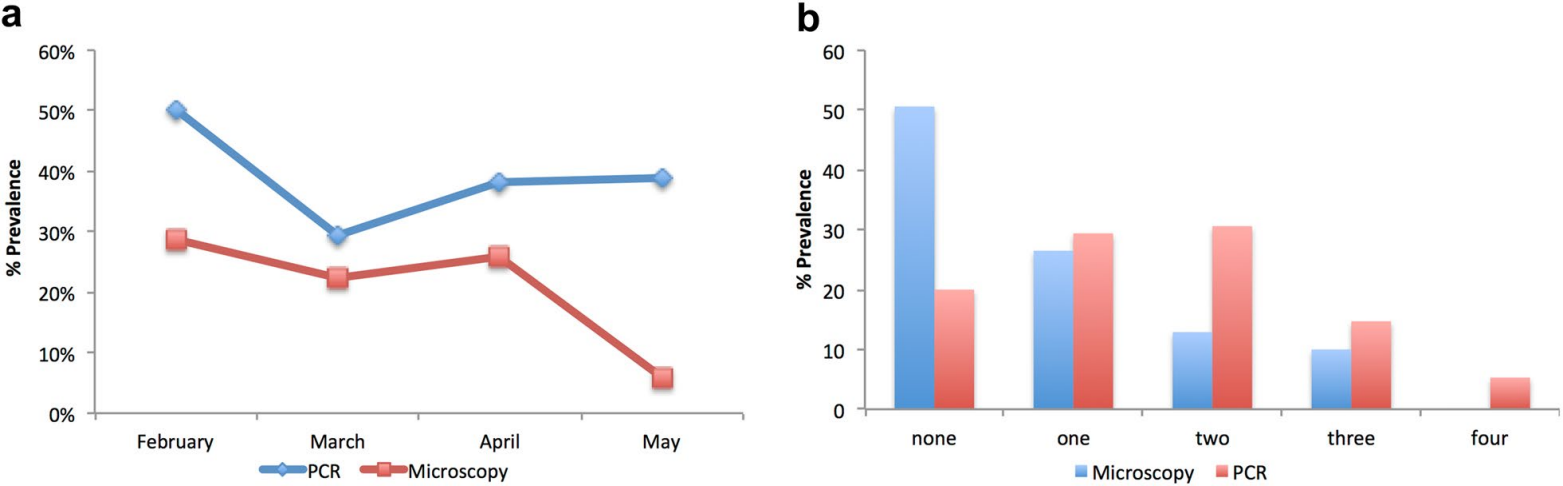
**a** *P. falciparum* parasite prevalence estimated by microscopy and PCR. Giemsa stained thick blood smears from samples collected monthly from a set of children were read and used to estimate parasite prevalence. PCR analysis of gDNA extracted from these same samples was also used to estimate parasite prevalence. **b** The number of times any particular child was classified as *P. falciparum* positive by either microscopy or PCR over the 4 month period was documented

The monthly *P. falciparum* prevalence estimated by PCR for February through May 2015 was 50 % (85/170), 29.4 % (50/170), 38.2 % (65/170) and 38.8 % (66/170) (Fig. 2a). PCR parasite genotyping identified 20 % (34/170) of the children to have remained parasite free during all the four visits, 29.4 % (50/170) to have been parasite positive at least once during the four visits, 30.6 % (52/170) to have had parasites on two visits, 14.7 % (25/170) to be parasite positive on three visits and 5.3 % (9/170) to have persistently carried parasites during each monthly visit (Fig. 1b).

Microscopy estimated *P. falciparum* prevalence for February through May 2015 as 28.8 % (49/170), 22.4 % (38/170), 25.9 % (44/170) and 5.9 % (10/170) (Fig. 2a). The proportion of children who remained parasite free by microscopy during all the four visits was 50.6 % (86/170), those that were positive at least once during the four visits was 26.5 % (45/170). The proportion of children who were parasite positive at two and three visits was 12.9 % (22/170) and 10 % (17/170) respectively. No child was identified as harbouring microscopic parasites at each of the four visits (Fig. 2b).

PCR detected a much higher prevalence of parasites than microscopy during each of the four survey points. Sub-microscopic *P. falciparum* carriage was estimated as the number of children with PCR detectable parasites who were classified as parasite negative by microscopy. Due to the much higher prevalence of parasites detected by PCR, sub-microscopic parasite carriage by the children during the survey period was generally high, especially in May when microscopic parasite carriage was the lowest.

### G6PD status and asymptomatic *Plasmodium falciparum* carriage

**Fig. 3:**
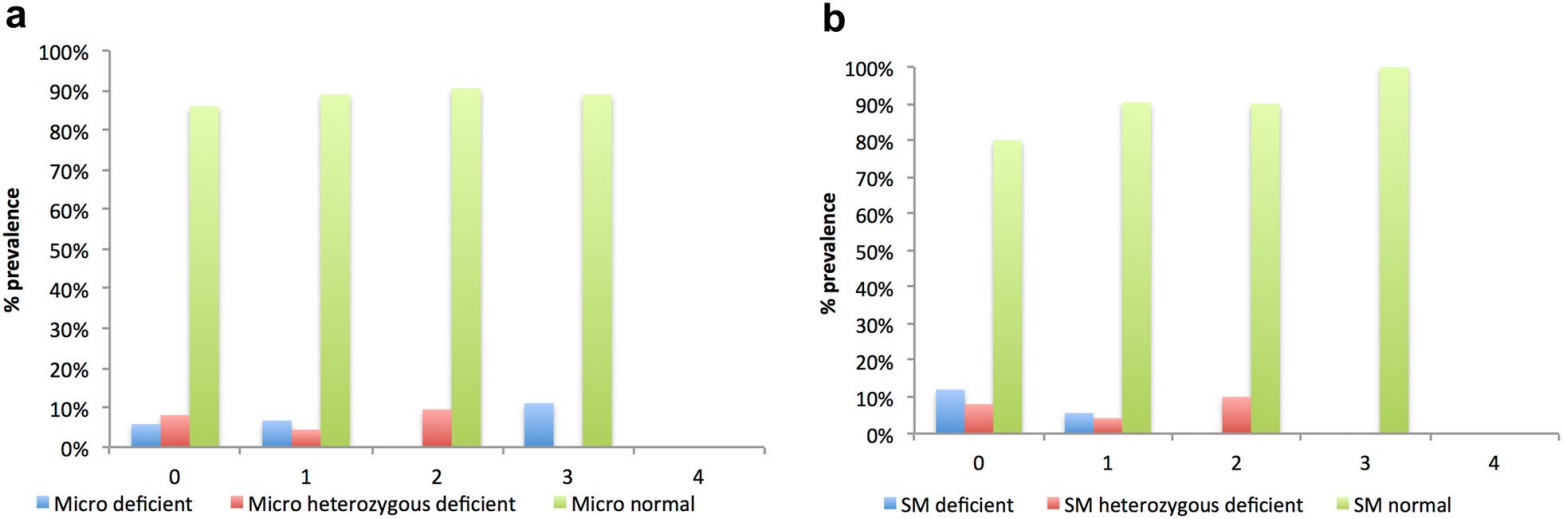
Frequency of *P. falciparum* carriage in G6PD normal and deficient children. Cumulative incidence of observed parasite carriage (microscopic and sub microscopic) in children classified as either G6PD normal or deficient by genotyping or RDT kit phenotypng over the 4 monthly visits. Frequency of microscopic (**a**) and sub microscopic parasite (**b**) parasite carriage within the three G6PD genotype groups, deficient (A- and A-/A-); Heterozygous deficient (A/A- or B/A-) and Normal (A, B, A/A, A/B, B/B)

Neither sub-microscopic parasite carriage nor microscopic parasite carriage over the 4 months was significantly associated with G6PD genotype and phenotype when tested with the model fit linear regression model (p > 0.05). However, parasite carriage estimated by PCR was significantly associated G6PD genotype but not G6PD phenotype (p < 0.05) (Table 3). There was a trend of a child having a lower tendency to carry malaria parasites at all four sampling times with both the G6PDd and the G6PDn children (Fig. 3a–d).

### Comparison of G6PDd prevalence by phenotypic RDT and PCR genotyping

Less than 5 % of children with normal G6PD genotypes were found to possess normal enzyme activity, however almost 50 % (44–56 %) of the hemi and homozygous deficient as well as the heterozygous deficient G6PD genotypes were found to possess normal enzyme activity using the qualitative G6PD RDT kits (Table 2).

**Table 2:** Analysis of G6PD genotype and phenotype

	Deficient (P)	Normal (P)
Deficient (G)	55.6 % (5/9)	44.4 % (4/9)
Heterozygous deficient (G)	44.4 % (4/9)	55.6 % (5/9)
Normal (G)	4.8 % (6/124)	95.2 % (118/124)

Deficient (P), deficient RDT read out; Normal (P), normal RDT read out; Deficient (G), deficient gene variant (A- and A-/A-); Heterozygous deficient (G), female with a heterozygous genotype (A/A- or B/A-); Normal (G), gene variants known to posses normal g6pd enzyme activity (A, B, A/A, A/B, B/B). All the samples were analysed for G6PD enzyme activity using the qualitative CareStart™ G6PD RDT kits as well as the presence of the sub Saharan African G6PD gene variants

## Discussion

In order to identify any possible influence of G6PD deficiency on asymptomatic malaria carriage, this study was conducted over the off peak malaria season, from February through to May 2015, when symptomatic *P. falciparum* carriage is low. Some previous studies have identified relationships between G6PD deficiency and protection from severe malaria [2, 31] as well as asymptomatic status [9]. In Ghana, however, most studies have concentrating on pregnant women due to the national sulfadoxine-pyrimethamine intermittent preventative treatment in pregnant women (SP-IPTp) policy [32–35]. Very few studies have focused on asymptomatic malaria patients or simultaneously carried out genotypic and phenotypic analysis of G6PD on the same individual [30, 36] as such, this study set out to find a possible correlation of G6PD with asymptomatic malaria in Ghana.

Asymptomatic parasite carriage estimated by PCR was very high, averaging about 40 % over the 4 months of the study (Fig. 2a). An average of 26 % of the children were identified as asymptomatic for malaria by microscopy for the first 2 months (February to April). The asymptomatic status of the children who tested positive for *P. falciparum* was confirmed by the fact that none of the children had a fever of 37.5 °C and above or any other physical symptoms of malaria during any of the four blood draws. Such high levels of asymptomatic *P. falciparum* carriage suggests that the children have developed immunity to malaria and serve as reservoirs for the parasite, which is likely to be a channel for intense malaria transmission when the mosquitoes start breeding during the rainy season.

The high prevalence (23 %) of samples, of children who were found to be parasite positive by microscopy more than once during the 4 monthly visits (Fig. 2b) could explain the sustained transmission of malaria immediately after the rainy season begins as there is a possible continuous production of gametocytes from these asexual parasites. The superiority of PCR over microscopy, the gold standard for the detection and diagnosis of malaria was confirmed in this study as the number of children found to be *P. falciparum* parasite free over the entire study period reduced from 50.6 % as observed by microscopy to 20 % after PCR analysis (Fig. 2b).

This study identified only the 376G/202A G6PDA- variant, which has so far been the only G6PD variant identified in Ghana [9, 30, 36]. The prevalence of G6PDd, of % (7/170) A- males and 1.8 % (3/170) A-/A- females identified in the study population (Table 1) is lower than previously published for Ghana [30]. The difference could be due to the use of a study population inhabiting the southern coast of Ghana as a previous report has shown that the prevalence of G6PDd in both males and females is higher in Kintampo, which is to the north of Kumasi than in Kumasi, which is in the middle belt of Ghana [30]. Forty four percent of G6PD A- and A-/A- children possed normal enzyme activity (Table 2) which is higher than that perviously reported in a study conducted in six African countrie including Ghana, where 10 % of G6PD A- males and 24 % G6PD A-/A- females possessed normal enzyme activity [30].

A previous study conducted in Ghana found G6PDd children to be about 1.5 times more likely to be parasitaemic based on microscopic evaluation of thin and thick blood films than non-deficient children [37], suggesting that the G6PD deficiency was associated with marginal susceptibility to clinical malaria in a child under 5 years of age. This study involved slightly older children who did not display such an association of G6PDd and malaria. PCR detectable parasite carriage but not parasite carriage by microscopy was significantly (p = 0.038) associated with the G6PD normal genotype (Table 3, Additional file 1), which slightly supports previous reports that suggests the G6PD trait offers some protection from malaria [38, 39]. G6PD deficient children also had a lower tendency to carry sub-microscopic *P. falciparum* parasites more than once during the 4 month period (Fig. 3a) although some G6PDd deficient children carried microscopically detectable parasites three out of the four sample visits (Fig. 3b).

**Table 3:** Summary of linear regression analysis

Model fit	Standardized coefficients^a^	Sig^a^. (PCR)	Standardized coefficients^b^	Sig^b^. (Micro)	Standardized coefficients^c^	Sig^c^. (SM)
	Beta (PCR)		Beta (Micro)		Beta (SM)	
(Constant)		0.032		0.009		0.103
G6PD (P)	−0.03	0.707	−0.011	0.89	0.126	0.112
G6PD (G)	0.166	0.038	0.02	0.8	0.133	0.093

G6PD (P), G6PD phenotype; G6PD (G), G6PD genotype

^a^Dependent variable: frequency of PCR detectable parasite carriage

^b^Dependent variable: frequency of Microscopic parasite carriage

^c^Dependent variable: frequency of sub microscopic parasite carriage

Genotyping G6PD by RFLP analysis identifies only the known G6PD genotypes, leaving the possibility of identifying novel mutations, which could possibly impact enzyme activity (28). A sample can be misclassified as normal due to the absence of particular mutations characterized despite carrying mutations that were not analysed. The six children who did not carry an A- allele but were identified as having reduced G6PD enzyme activity (Table 2) could have possessed other deficiency causing mutations that were not characterized possibly because they have not been assigned a genotype [40]. A study conducted in the Gambia on 1437 children between 5 and 14 years found half (50 %) the study population to carry the G6PD A376G (B) variant and 39 % to carry mutations that have not been assigned a genotype [41]. Heterozygous G6PD deficient females are able to randomly inactivate one of their two X-chromosomes and as such exhibit either normal or deficient G6PD enzyme activity [42, 43]. As such a little over half (5/9) of the heterozygous deficient children exhibited normal G6PD enzyme activity.

The CareStart™ G6PD RDT classified 44.4 % (4/9) of A- and A-/A- deficient children as normal (to possess normal G6PD enzyme activity). This could be due to inaccuracies and the level of subjectivity of the RDT kit readout [13, 14, 16, 44]. Some studies have found people with the same G6PD cDNA mutation to exhibit different enzyme activities due to possible differences in the G6PD acetylator in the deficient persons [45] as well as the possible contribution of a number of superimposed genetic deficiencies [46] and blood sugar levels [47].

## Limitations

Genetic analysis of G6PD deficiency was carried out using RFLP and specifically determined common sub-Saharan African G6PD genotypes. As such other deficiency causing mutations which were not analysed could have been present in the study population. Additional characterization of red blood cells were not performed although certain red blood cell conditions such as anaemia, which causes a reduction in the volume of older G6PDd RBC’s and an increase in reticulocytes, could result in anaemic G6PDd child exhibiting near normal G6PD enzyme activity [48]. A disadvantage to the rapid qualitative CareStart™ G6PD RDT kit used for G6PD phenotypic analysis is that read out can be subjective and a few sample results could have been misinterpreted.

## Conclusions

Asymptomatic *P. falciparum* carriage over the off peak malaria season of 30 to 50 % could likely be a driving force to sustained malaria transmission over the off peak season. Twelve percent of the school children carried genetically deficient G6PD genes and 11 % were identified as having deficient G6PD enzyme activity. The frequency of parasite carriage estimated by PCR was significantly associated with G6PD phenotype. A similar study should be conducted in the peak malaria season to determine the consequences asymptomatic *P. falciparum* carriage has on malaria transmission as well as identify association between G6PD and malaria in these same children during the peak season.

## Additional file

10.1186/s12936-016-1440-1 Additional tables.
