# Supplementary material for: Prevalence of G6PD deficiency and Plasmodium falciparum parasites in asymptomatic school children living in southern Ghana
Source: Malar J. 2016 Jul 26;15:388. doi: 10.1186/s12936-016-1440-1 (PMC4960760; doi:10.1186/s12936-016-1440-1)
Supplement: Supplementary file 1 — 10.1186/s12936-016-1440-1 Additional tables. [file 12936_2016_1440_MOESM1_ESM.docx]

| **Coefficients^a^** | | | | | | |
| --- | --- | --- | --- | --- | --- | --- |
| Model | | Unstandardized Coefficients | | Standardized Coefficients | t | Sig. |
|  |  | B | Std. Error | Beta |  |  |
| 1 | (Constant) | .879 | .407 |  | 2.160 | .032 |
|  | G6PD (P) | -.099 | .262 | -.030 | -.377 | .707 |
|  | G6PD (G) | .457 | .219 | .166 | 2.092 | .038 |
| a. Dependent Variable: frequency of PCR parasite carriage | | | | | | |

| **Coefficients^a^** | | | | | | |
| --- | --- | --- | --- | --- | --- | --- |
| Model | | Unstandardized Coefficients | | Standardized Coefficients | t | Sig. |
|  |  | B | Std. Error | Beta |  |  |
| 1 | (Constant) | .771 | .291 |  | 2.653 | .009 |
|  | G6PD (P) | -.026 | .187 | -.011 | -.139 | .890 |
|  | G6PD (G) | .040 | .156 | .020 | .254 | .800 |
| a. Dependent Variable: frequency of Microscopic parasite carriage | | | | | | |

| **Coefficients^a^** | | | | | | |
| --- | --- | --- | --- | --- | --- | --- |
| Model | | Unstandardized Coefficients | | Standardized Coefficients | t | Sig. |
|  |  | B | Std. Error | Beta |  |  |
| 1 | (Constant) | .420 | .256 |  | 1.638 | .103 |
|  | G6PD (P) | .263 | .165 | .126 | 1.597 | .112 |
|  | G6PD (G) | .233 | .138 | .133 | 1.691 | .093 |
| a. Dependent Variable: frequency of Sub Microscopic parasite carriage | | | | | | |
